# Impact of systemic inflammation and disease activity on the incidence of interstitial lung disease in patients with rheumatoid arthritis – a nested case-control study within the German biologics register RABBIT

**DOI:** 10.1186/s13075-024-03449-9

**Published:** 2024-12-05

**Authors:** Ronja Ramien, Tatjana Rudi, Rieke Alten, Andreas Krause, Matthias Schneider, Martin Schaefer, Anja Strangfeld, Yvette Meissner

**Affiliations:** 1 Epidemiology and Health Services Research, German Rheumatology Research Center, Charitéplatz 1, 10117 Berlin, Germany; 2 Privataerztliches Zentrum am Roseneck, Berlin, Germany; 3 Schlosspark-Klinik Charlottenburg, Berlin, Germany; 4 Immanuel Krankenhaus, Berlin, Germany; 5 Heinrich-Heine-University, Dusseldorf, Germany; 6 Department of Rheumatology and Clinical Immunology, Charité University Medicine, Berlin, Germany

**Keywords:** Disease activity, Epidemiology, Incidence, Inflammation, Interstitial lung disease, Rheumatoid arthritis, Risk factors

## Abstract

**Background:**

To investigate the association between the development of incident interstitial lung disease (ILD) in patients with rheumatoid arthritis (RA) and the disease activity of RA with its various components, especially C-reactive protein (CRP) and erythrocyte sedimentation rate (ESR).

**Methods:**

We analysed data from RA patients, observed in the German biologics register RABBIT between 2001 and 2021. In a nested case-control study, patients with a reported incident ILD diagnosis during follow-up were matched 1:5 to patients without ILD. Matching criteria were sex, age, RA duration, date of enrolment and observation time. Defined by a directed acyclic graph (DAG), we adjusted the conditional logistic regression models for rheumatoid factor, smoking, chronic obstructive pulmonary disease and tuberculosis/chronic viral infections to investigate the impact of disease activity/systemic inflammation. Mean and categorized values were analysed within 12 months prior to ILD and during the entire observation time. Additionally, two sensitivity analyses were performed, using validated ILD cases only and considering ILD cases with an observation time of more than 12 months.

**Results:**

We identified 139 RA patients with incident ILD and matched them to 686 controls. In 94 cases the diagnosis could be validated, and 98 cases had a follow-up of > 12 months. The averaged DAS28 composite score (including ESR or CRP) was not associated with developing RA-ILD (odds ratios 1.16 [95% confidence interval: 0.97–1.40] and 1.06 [0.86–1.29], respectively). However, single measures of inflammation, log ESR (1.86 [1.35–2.57]) and log CRP (1.55 [1.21–1.97]), were significantly associated with an increased RA-ILD risk. A higher risk for ILD was also revealed for persistently high inflammation. Other DAS28 components showed no significant associations with RA-ILD. These results were consistent for values over the entire observation time of a patient and within 12 months prior to the ILD. Sensitivity analyses confirmed these findings.

**Conclusion:**

Higher levels of systemic inflammation, as indicated by ESR and CRP, but not joint counts or patient’s global assessment, were significantly associated with the occurrence of incident ILD in patients with RA. As possible predictor for the development of RA-ILD, systemic inflammation should be monitored closely and independently of joint count results.

**Supplementary Information:**

The online version contains supplementary material available at 10.1186/s13075-024-03449-9.

## Background

Rheumatoid arthritis (RA) is a chronic systemic inflammatory disease with a lifetime risk of 3.6% in women and 1.1% in men [1]. While inflammation in the joints is the main manifestation of RA, extra-articular manifestations are common. They can occur, e.g., in the lungs in the form of interstitial lung diseases (ILD) or other manifestations [2]. Up to 10% of the patients with RA develop an ILD (RA-ILD) [3], which is associated with higher morbidity and mortality [4, 5]. The risk for patients with RA to develop an ILD is about nine times higher compared to the general population [6].

Interstitial lung diseases are a heterogeneous group of about 200 conditions with a different pathogenesis characterized by inflammation and fibrosis of the pulmonary interstitium [7]. They can be classified into ILDs with ascertainable causes and idiopathic ILDs without identifiable causes [8]. In case of RA-ILD, the associated pulmonary manifestation is linked to the underlying autoimmune disease, yet the exact pathomechanism remains unclear [9]. Symptoms are frequently non-specific and include respiratory complaints like dry cough, dyspnea or chest pain [10]. However, up to 55% of RA-ILD patients do not show any respiratory symptoms at all [11, 12]. The diagnostic workup includes blood tests, lung function tests, bronchoalveolar lavage (BAL) and X-ray, even though these methods are not sensitive and specific enough [8]. Nowadays, the preferred diagnostic method is therefore high-resolution computed tomography (HRCT) [13].

Certain risk factors for the development of ILD in general and RA-ILD in particular are known so far. In general, males, smokers and patients with specific comorbidities such as chronic obstructive pulmonary disease, history of hepatitis C, pneumonia or tuberculosis have a higher risk for incident ILD [14]. In RA patients, age above 55 years at onset of the RA, long disease duration, and destruction of the joints are associated with an increased risk for RA-ILD [3, 15]. Furthermore the presence of rheumatoid factor (RF) and/or anti-citrullinated protein antibodies (ACPA), a genetic predisposition, e.g., MUC5B promoter variant rs35705950, high levels of matrix metalloproteinase-3 and other extra-articular manifestations of RA like subcutaneous rheumatoid nodules or secondary systemic sclerosis show an association with an increased risk for RA-ILD [15–17]. The impact of disease-modifying antirheumatic drugs (DMARDs), especially methotrexate (MTX) [18], leflunomide [19] or tumour necrosis factor inhibitors (TNFi) [20], on the development of RA-ILD is a subject of ongoing research, although causality has not yet been clearly demonstrated [21].

Several studies have identified elevated or moderate disease activity as a contributing factor for RA-ILD [22–25]. Of note, most of the studies only used values from one single time point, e.g., study enrolment or ILD onset. In terms of systemic inflammation, results are inconclusive. Whilst some studies showed no significant association between C-reactive protein (CRP) and/or erythrocyte sedimentation rate (ESR) levels and ILD incidence [22, 26], others have demonstrated an impact of either elevated levels of CRP [16, 23, 24] or ESR [27–29]. To date, only one study has methodically focused on disease activity as a risk factor for incident ILD in RA patients. They also focused on the inflammatory marker CRP, but did not include ESR [22].

The primary objective of our study was to examine the relationship between incident RA-ILD and the Disease Activity Score based on 28 tender and swollen joint count (DAS28), with a particular emphasize on its individual components, especially the systemic inflammation markers ESR and CRP. Secondary objectives comprised the investigation of ILD incidence and treatment with DMARDs prior to ILD diagnosis.

## Patients and methods

### Data source and patient population

We used data from patients with RA enrolled and observed in the RABBIT register (Rheumatoid Arthritis: Observation of Biologic Therapy), an ongoing Germany-wide prospective longitudinal cohort study initiated in 2001 with the aim to investigate the long-term safety and effectiveness of different DMARD treatments. Adult patients with RA are eligible for enrolment when either starting a biologic (b)DMARD or a targeted synthetic (ts)DMARD treatment, or a conventional synthetic (cs)DMARD drug after at least one prior DMARD treatment. Data is reported by rheumatologists and patients via questionnaires at enrolment and at fixed time points of follow-up (at month three and six, and then every six months). At enrolment, information is collected on characteristics of the disease, e.g., RA duration and activity, RF status, comorbidities and prior DMARD treatments. Regularly collected information includes disease measures, antirheumatic treatment details and the occurrence of (serious) adverse events ((S)AEs). In case of SAEs, rheumatologists are requested to provide hospital discharge letters. All adverse events (AEs) are coded using the Medical Dictionary for Regulatory Activities (MedDRA) on the preferred term level. Patients are followed for at least five and up to ten years. Study details are provided elsewhere [30]. The original study protocol was approved in May 2001 by the ethics committee of the Charité University Medicine Berlin, Germany, and an updated revision received approval in July 2021 (EA4/123/21). All patients have to give written informed consent prior to enrolment.

For this study, patients enrolled between May 2001 and October 2021 with at least one follow-up observation were selected. Patients with a prevalent ILD reported as comorbidity at enrolment and patients with missing information on pulmonary comorbidities or with inconsistent statements regarding pulmonary comorbidities during follow-up were excluded.

### Outcome definition

All reported SAEs and AEs were screened for terms included in the Standardised MedDRA Query “interstitial lung disease”. Out of those, events that were not covered by ILD codes of the International Classification of Diseases, 10th Revision (ICD-10) were excluded (supplementary Table 1) [31]. These comprised the following diagnoses: pulmonary granuloma, acute respiratory distress syndrom (not caused by ILD), acute lung injury, lung infiltration, pulmonary rheumatoid nodules, pulmonary radiation injury, granulomatosis with polyangiitis and sarcoidosis.

Discharge letters of all ILD events were screened to validate the exact diagnosis, the date of diagnosis and the diagnostic method, categorized into computed tomography (CT), x-ray and other methods. If no discharge letter was available, the reporting rheumatologist was contacted for event validation using a structured questionnaire.

### Study design

A nested case-control study was performed in which patients in whom ILD was reported as an incident event during the observation period were defined as cases. Each case was matched at a ratio of 1:5 to controls (1 case and 5 controls formed a cluster). Controls were defined as patients who did not experience an ILD throughout the entire observation period. Matching criteria were sex, age (+/- 5 years), RA duration (+/-3 years), date of enrolment in RABBIT (+/- 2 years) and observation time. For cases, the index date was defined as the date of ILD diagnosis (supplementary Fig. 1). For controls, the index date was a chosen date resulting in the same observation time as the corresponding case up to their corresponding index date. The matching was applied using the R-package Optmatch of the freely available software R [32].

### Exposure definition

Disease activity, measured by DAS28-ESR and DAS28-CRP, and systemic inflammation, measured by ESR and CRP, were studied as continuous and categorized variables. Moderate to high disease activity was defined as DAS28-ESR ≥ 3.2 and DAS28-CRP ≥ 2.9, and high systemic inflammation as ESR > 21 mm/h and CRP ≥ 5 mg/L. Furthermore, other DAS28 components were evaluated, i.e., patient’s global health and the 28-joint count on swollen and tender joints.

### Statistical analysis

Patient characteristics are presented using descriptive statistics (numbers, percentages, means, standard deviations). To examine the incidence of ILD over time, the number of incident events occurring in one year was divided by the total number of patients “at risk” at the beginning of that year. Incidences are depicted in a scatter plot, and a fitted regression curve illustrates the incidence trend over time. The correlation between ILD incidence and time was determined by Spearman and Kendall tau-b correlation coefficients.

Antirheumatic treatment was analysed by stratifying DMARDs into csDMARDs, TNFi, interleukin-6 inhibitors (IL6i), T-cell co-stimulation modulator (T-cell), B-cell-targeted therapy (B-cell) and Janus kinase inhibitors (JAKi). A descriptive analysis investigated DMARDs ever received until the index date. Further, the number of different b/tsDMARDs in cases and controls was compared within (a) 12 months prior to the index date and (b) the entire observation time from enrolment until the index date by mixed-effects Poisson regression models with a random component for each cluster.

To visualize the course of disease activity and systemic inflammation, mean and categorized values were mapped (a) in the 12 months prior to the index date and (b) in the first 12 months after enrolment by mixed models using the cluster as random effect to calculate mean values including their respective confidence intervals (CI).

The impact of DAS28 and its individual components on ILD incidence was assessed by multivariable conditional logistic regression; unadjusted regression models are shown in supplementary Table 2. In the main model, all cases meeting the ILD definition were analysed (see outcome definition). Odds-ratios (OR) and 95% CI were estimated for continuous values (a) as means within 12 months prior to the index date and (b) as means throughout the entire observation period (enrolment until index date). In addition, DAS28 and inflammation markers were investigated by categorizing the values for each month according to the cut-offs described in the exposure definition. To calculate the proportion of months with elevated inflammatory disease activity, the number of months with elevated values was counted and divided by the total observation time in months [for (a) 12 months prior to the index date and (b) the entire observation time]. Regression models were adjusted for the following covariates identified by a directed acyclic graph (DAG, drawn with the program DAGitty [33], see supplementary Fig. 2): RF, smoking (ever/current vs. never), chronic obstructive pulmonary disease, tuberculosis and/or chronic viral infections (hepatitis B, hepatitis C, human immunodeficiency virus). Information on covariates was used from the entire observation time up to the index date except for RF and smoking, which were assessed at enrolment. We have refrained from including therapies in our models as there is no proven association between DMARDs and the development of ILD, and therefore DMARDs were not identified as confounders in our DAG. Further, we added matching variables as covariates to the regression models to consider possible residual confounding and possible selection bias introduced by case-control matching on a confounder as explained by Mansournia et al. [34]. Models without adjustment for matching variables are shown in supplementary Table 3. Missing values of exposure and confounders (supplementary Table 4) were imputed 10 times by multiple imputation using the fully conditional specification method [35]. For regression analyses, CRP and ESR were log-transformed due to their skewed distribution.

Two sensitivity analyses were performed: (I) the regression analysis was restricted to cases with a validated ILD diagnosis and (II) case-control clusters with an observation time of 12 months or less were excluded. To account for current recommendations [36, 37], cases diagnosed by x-ray were excluded from sensitivity analysis I in a further step.

In regression models, *P* values < 0.05 were considered statistically significant. Data analyses were performed with SAS V.9.4 (SAS Institute, Cary NC).

## Results

**Fig. 1:**
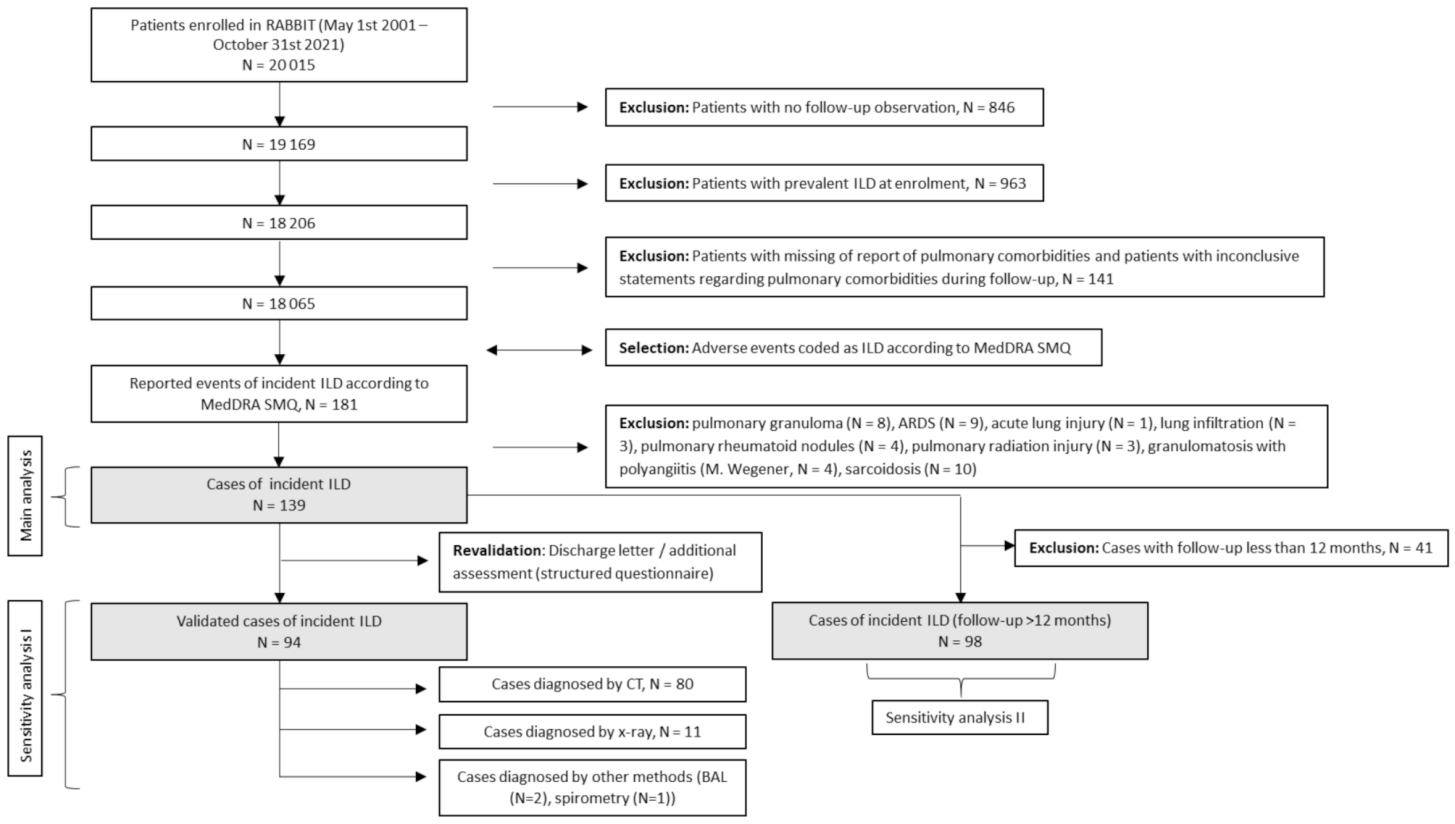
Flow chart for patient selection Abbreviations: ARDS: acute respiratory distress syndrome; BAL: bronchoalveolar lavage; CT: computed tomography; ILD: interstitial lung disease; MedDRA: medical dictionary for regulatory activities; SMQ: standardised MedDRA queries

Until October 2021, a total of 20 015 patients with RA were enrolled into the RABBIT register (Fig. 1).

After applying the exclusion criteria, 18 065 patients were eligible for analysis. Out of these, 139 cases of incident ILD were identified (main analysis) and matched 1:5 to 686 controls without ILD. However, 1:5 matching was not feasible for five cases, with two being matched at 1:2 and three at 1:4. Characteristics of respective cases are provided in supplementary Table 5. In 94 cases, events could be validated by discharge letters (sensitivity analysis I). Thereof, 80 ILDs were diagnosed by CT, eleven by x-ray and three by other methods (BAL (*n* = 2), spirometry (*n* = 1)). For sensitivity analysis II, 41 cases with follow-up observation of less than 12 months were excluded, resulting in the investigation of 98 cases.

### Patient characteristics

Cases and their matched controls were comparable in matching criteria: two thirds of cases and controls were female, mean age was 61 to 62 years, disease duration 9 to 10 years and mean observation time 38 months (Table 1). Agreement was also reached on the year of enrolment. In terms of characteristics not matched for, cases presented with similar DAS28 scores but tended to have higher levels of systemic inflammation compared to controls. Cases showed a higher frequency of RF positivity, rheumatoid nodules, smoking habit and comorbidities. Differences were also observed in antirheumatic treatments started at enrolment: Cases were less often treated with TNFi and more often with T-cell or B-cell therapy than matched controls.

**Table 1:** Enrolment characteristics of cases (patients who developed an interstitial lung disease during follow up), their matched controls and remainders of the RABBIT cohort

	Cases *N* = 139	Controls *N* = 686	Remainders of the cohort *N* = 17 240
**Matching criteria**			
Sex, female	90 (64.7)	441 (64.3)	13 072 (75.8)
Age (years), mean (SD)	62.2 (10.7)	61.4 (10.4)	56.5 (12.8)
RA duration (years), mean (SD)	9.6 (9.4)	9.3 (8.9)	9.3 (8.8)
Year of enrolment 2001–2005	39 (28.1)	192 (28.0)	3 623 (21.0)
Year of enrolment 2006–2010	37 (26.6)	177 (25.8)	3 690 (21.4)
Year of enrolment 2011–2015	39 (28.1)	196 (28.6)	4 491 (26.1)
Year of enrolment 2016–2021	24 (17.3)	121 (17.6)	5 436 (31.5)
Observation time (months), mean (SD)	38.0 (33.0)	37.5 (32.7)	57.5 (39.1)
**Unmatched criteria**			
Age at RA onset (years), mean (SD)	52.6 (13.6)	52.1 (13.3)	47.2 (14.1)
DAS28-ESR, mean (SD)	5.2 (1.4)	5.1 (1.3)	5.0 (1.4)
DAS28-CRP, mean (SD)	4.0 (1.1)	3.9 (1.1)	3.8 (1.1)
ESR (mm/hour), mean (SD)	34.8 (21.6)	29.5 (21.8)	28.7 (22.4)
CRP (mg/L), mean (SD)	19.4 (24.2)	17.0 (22.5)	16.3 (23.9)
Tender joints, mean (SD)	8.9 (7.0)	8.6 (7.0)	8.2 (6.7)
Swollen joints, mean (SD)	7.1 (6.0)	6.5 (5.3)	5.8 (5.2)
Patient’s global health, mean (SD)	5.8 (2.2)	5.8 (2.0)	5.8 (2.1)
% of full physical function (FFbH), mean (SD)	62.2 (25.5)	63.4 (22.8)	65.5 (23.1)
Rheumatoid factor positive	114 (82.0)	496 (72.3)	11 779 (68.3)
Rheumatic nodules	40 (28.8)	98 (14.3)	2 665 (15.5)
Erosions of joints	52 (37.4)	266 (38.8)	7 715 (44.8)
Smoking, ever	83 (59.7)	339 (49.4)	8 706 (50.5)
Smoking, never	50 (36.0)	319 (46.5)	7 312 (42.4)
Number of comorbidities*, mean (SD)	3.4 (2.9)	2.5 (2.4)	2.1 (2.1)
Chronical viral infection^#^	2 (1.4)	2 (0.3)	57 (0.3)
Chronic obstructive pulmonary disease	12 (8.6)	45 (6.6)	757 (4.4)
Coronary heart disease	21 (15.1)	55 (8.0)	1 080 (6.3)
Chronic kidney disease	14 (10.1)	38 (5.5)	825 (4.8)
Arterial hypertension	86 (61.9)	318 (46.4)	6 823 (39.6)
Osteoporosis	42 (30.2)	142 (20.7)	2 865 (16.6)
Latent tuberculosis	6 (4.3)	13 (1.9)	267 (1.5)
BMI ≥ 30 kg/m^2^	43 (30.9)	170 (24.8)	4 227 (24.5)
Enrolment therapy: csDMARD	44 (31.7)	243 (35.4)	5 245 (30.4)
Enrolment therapy: TNFi	61 (43.9)	323 (47.4)	8 348 (48.5)
Enrolment therapy: T-cell	9 (6.5)	19 (2.8)	628 (3.6)
Enrolment therapy: B-cell	13 (9.4)	31 (4.5)	896 (5.2)
Enrolment therapy: IL6i	8 (5.8)	53 (7.7)	1243 (7.2)
Enrolment therapy: JAKi	4 (2.9)	15 (2.2)	870 (5.0)
Count of prior b/tsDMARD, mean (SD)	0.4 (0.8)	0.3 (0.8)	0.3 (0.8)
Glucocorticoids, < 5 mg/day	58 (41.7)	300 (43.7)	7 981 (46.3)
Glucocorticoids, 5–10 mg/day	58 (41.7)	269 (39.2)	6 247 (36.2)
Glucocorticoids, > 10 mg/day	22 (15.8)	115 (16.8)	2 794 (16.2)

Values are numbers of patients (%) unless otherwise specified

Abbreviations: B-cell: B-cell-targeted therapy, bDMARD: biologic disease-modifying antirheumatic drug, BMI: body mass index, CRP: C-reactive protein, csDMARD: conventional synthetic disease-modifying antirheumatic drug, DAS28: disease activity score based on 28 tender and swollen joint count, ESR: erythrocyte sedimentation rate, FFbH: Funktionsfragebogen Hannover, IL-6i: Interleukin-6 inhibitors, JAKi: Janus kinase inhibitors, RA: rheumatoid arthritis, SD: standard deviation. T-cell: T-cell co-stimulation modulator, TNFi: tumour necrosis factor inhibitors, tsDMARD: targeted synthetic disease-modifying antirheumatic drug

*Comorbidities: arterial hypertension, coronary heart disease, heart failure, cerebral ischemia, hypolipoproteinaemia, diabetes, chronic kidney disease, chronic viral infection (#hepatitis B, hepatitis C, human immunodeficiency virus), chronic liver disease, asthma bronchiale, ulcus duodeni/ ventriculi, chronic obstructive lung disease, other gastrointestinal diseases, degenerative spine disease, degenerative joint disease, osteoporosis, Sjoegren syndrome, psoriasis, fibromyalgia, psychological disorders, latent tuberculosis, lymphoma/leukaemia, malignant neoplasia

Table 1 additionally presents characteristics of the “remainders”, 17 240 patients in RABBIT that were neither cases nor selected as controls. Despite a similar disease duration as the cases and controls, the remaining patients were more often female, on average 5 years younger, and had a longer observation period of 57 months. For most of the unmatched criteria, the cohort remainders were similar to control patients.

### Incidence of RA-ILD

The cumulative incidence of RA-ILD in the RABBIT cohort was 0.8%. The incidence proportion varied over time between 0.03% and 0.28% per year. No significant trend in incidence proportion was observed between 2003 and 2021 (supplementary Fig. 3).

### Antirheumatic treatment

Cases and matched controls differed in the DMARD treatments they had ever received. At the index date, cases were less often bionaïve with 20.1% compared to 25.9% in controls (Table 2). Stratifying patients by the number of b/tsDMARDs with different modes of action until the index date, there were more patients with at least two b/tsDMARDs in cases than in matched controls (36.7% vs. 25.4%). Furthermore, the frequencies of b/tsDMARD use differed. Cases had more often received T-cell and B-cell therapy as first bDMARD therapy (5.0% for T-cell and B-cell therapy each vs. 1.5% each in controls), and in patients with two different b/tsDMARDs, the percentage of B-cell therapy was higher (44.4% vs. 32.3% in controls).

**Table 2:** Antirheumatic treatment with DMARDs ever received until index date stratified by number of b/tsDMARD mode of actions

DMARD at the index date	Cases *N* = 139					Controls *N* = 686				
csDMARD only (bionaive)	20.1%					25.9%				
1 b/tsDMARD mode of action	43.2%					48.5%				
	TNFi 86.7%	B-cell 5.0%	T-cell 5.0%	IL6i 0%	JAKi 3.3%	TNFi 87.8%	B-cell 1.5%	T-cell 1.5%	IL6i 5.7%	JAKi 3.6%
2 b/tsDMARD* mode of actions	25.9%					17.6%				
	Others 8.3%	TNFi-B-cell 44.4%	TNFi-T-cell 11.1%	TNFi-IL6i 30.6%	TNFi-JAKi 5.6%	Others 5.5%	TNFi-B-cell 32.3%	TNFi-T-cell 16.6%	TNFi-IL6i 39.8%	TNFi-JAKi 5.8%
3 b/tsDMARD* mode of actions	6.5%					5.2%				
4 b/tsDMARD* mode of actions	3.6%					2.0%				
5 b/tsDMARD* mode of actions	0.7%					0.6%				

*Order of b/tsDMARDs may vary

Abbreviations B-cell: B-cell-targeted therapy, bDMARD: biologic disease-modifying antirheumatic drug, csDMARD: conventional synthetic disease-modifying antirheumatic drug, IL6i: Interleukin-6 inhibitors, JAKi: Janus kinase inhibitors, T-cell: T-cell co-stimulation modulator, TNFi: tumor necrosis factor inhibitors, tsDMARD: targeted synthetic disease-modifying antirheumatic drug

Restricting the investigation of b/tsDMARD use to the year prior to the index date, cases tended to have received more different treatments compared to controls (Poisson regression: 1.12 [95% CI 0.91–1.36]). Regarding the entire observation time from enrolment until index date, the result was similar (1.14 [0.96–1.36]).

### Course of disease activity and systemic inflammation

**Fig. 2:**
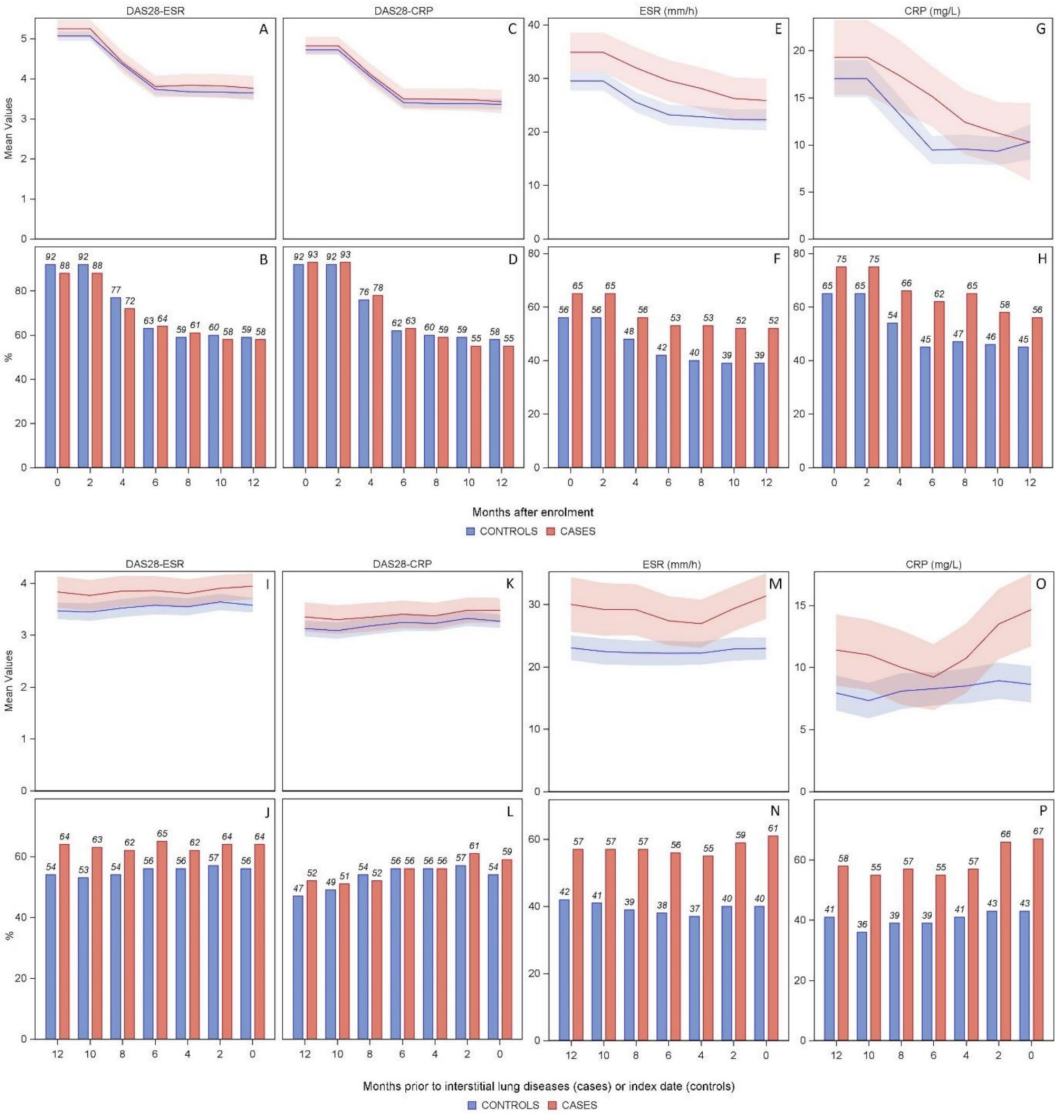
Disease activity and systemic inflammation in the 12 months after enrolment and in the 12 months prior to the index date Line charts(A, C, E, G, I, K, M, O): Mean values of DAS28-ESR, DAS28-CRP, ESR and CRP, including 95% confidence bands of cases with interstitial lung disease (red) and matched controls (blue) Bar charts (B, D, F, H, J, L, N, *P*): Percentage of patients with DAS28-ESR ≥ 3.2, DAS28-CRP ≥ 2.9, ESR > 21 mm/h and CRP ≥ 5 mg/L of cases with interstitial lung disease (red) and matched controls (blue) Abbreviations: CRP: C-reactive protein, DAS28: disease activity score based on 28 tender and swollen joint count, ESR: erythrocyte sedimentation rate

Cases showed significantly higher levels of systemic inflammation compared to matched controls (Fig. 2) during observation. However, disease activity levels were comparable between the two groups. This is not only evident within the 12 months prior to the index date, but also within the first 12 months after enrolment. Furthermore, cases were more often in a high inflammatory status within 12 months prior to the index date with 57–61% having ESR levels > 21 mm/h (Fig. 2, N) and 55–67% having CRP levels ≥ 5 mg/L (Fig. 2, P). Respective frequencies in controls were 37–42% for ESR and 36–43% for CRP. Differences in moderate to severe disease activity were not as distinct, especially not for DAS28-CRP.

### Association between disease activity, systemic inflammation and incident ILD

Disease activity as measured by DAS28-ESR and DAS28-CRP was not significantly associated with the occurrence of RA-ILD throughout the observation time, neither as mean value nor as categorized value (Table 3). In contrast, both inflammation markers showed a significant association with RA-ILD. The OR (95% CI) for log ESR was 1.86 (1.35–2.57) and for log CRP 1.55 (1.21–1.97), both calculated as mean values for the entire observation period. This association was more pronounced when analysing categorized values: having ESR levels higher than 21 mm/h throughout the entire time until index date showed an OR of 2.98 (1.72–5.17) compared to levels ≤ 21 mm/h, and CRP ≥ 5 mg/L showed an OR of 3.70 (1.95–6.89) compared to levels lower than 5 mg/L. Neither swollen and tender joint count nor patient’s global health assessment were significantly associated with the incidence of RA-ILD. Restricting the analysis to the 12 months prior to the index date showed comparable results. However, the point estimate of mean DAS28-ESR reached statistical significance (OR 1.21 [95% CI 1.03–1.43]).

**Table 3:** Results of adjusted multivariable logistic regression analysis for the chance of developing interstitial lung disease

	Main analysis	Sensitivity analyses	
		I	II
	OR (95% CI)	OR (95% CI)	OR (95% CI)
**Within 12 months prior to index date**			
Mean DAS28-ESR	1.21 (1.03–1.43)	1.26 (1.02–1.55)	1.30 (1.06–1.60)
DAS28-ESR ≥ 3.2 vs. DAS28-ESR < 3.2*	1.41 (0.83–2.42)	1.41 (0.74–2.69)	1.69 (0.94–3.05)
Mean DAS28-CRP	1.11 (0.92–1.34)	1.16 (0.92–1.46)	1.24 (0.99–1.56)
DAS28-CRP ≥ 2.9 vs. DAS28-CRP < 2.9*	1.06 (0.62–1.80)	1.23 (0.65–2.35)	1.22 (0.68–2.20)
Mean Log ESR	1.72 (1.30–2.27)	1.72 (1.24–2.39)	1.59 (1.15–2.20)
ESR > 21 mm/h vs. ESR ≤ 21 mm/h*	2.58 (1.58–4.21)	2.55 (1.41–4.62)	2.21 (1.20–4.05)
Mean Log CRP	1.51 (1.20–1.88)	1.48 (1.14–1.93)	1.56 (1.17–2.08)
CRP ≥ 5 mg/L vs. CRP < 5 mg/L*	2.86 (1.70–4.82)	2.58 (1.41–4.73)	2.73 (1.48–5.05)
Mean patient’s global health	0.95 (0.86–1.06)	0.97 (0.85–1.10)	0.97 (0.86–1.10)
Mean swollen joint count	1.02 (0.96–1.07)	1.02 (0.95–1.10)	1.02 (0.94–1.10)
Mean tender joint count	1.01 (0.97–1.05)	1.02 (0.97–1.07)	1.05 (1.00-1.10)
**Total observation until the index date**			
Mean DAS-ESR	1.16 (0.97–1.40)	1.25 (0.99–1.56)	1.25 (0.99–1.57)
DAS28-ESR ≥ 3.2 vs. DAS28-ESR < 3.2*	1.22 (0.62–2.42)	1.32 (0.57–3.07)	1.62 (0.73–3.60)
Mean DAS28-CRP	1.06 (0.86–1.29)	1.14 (0.89–1.46)	1.18 (0.92–1.52)
DAS28-CRP ≥ 2.9 vs. DAS28-CRP < 2.9*	1.04 (0.54–2.03)	1.22 (0.54–2.75)	1.36 (0.63–2.93)
Mean Log ESR	1.86 (1.35–2.57)	1.83 (1.26–2.68)	1.71 (1.15–2.54)
ESR > 21 mm/h vs. ESR ≤ 21 mm/h *	2.98 (1.72–5.17)	2.71 (1.41–5.20)	2.53 (1.24–5.19)
Mean Log CRP	1.55 (1.21–1.97)	1.52 (1.14–2.02)	1.65 (1.18–2.30)
CRP ≥ 5 mg/L vs. CRP < 5 mg/L*	3.70 (1.95–6.89)	3.02 (1.47–6.21)	3.88 (1.73–8.67)
Mean patient’s global health	0.94 (0.83–1.06)	0.94 (0.82–1.08)	0.96 (0.83–1.11)
Mean swollen joint count	1.02 (0.96–1.08)	1.04 (0.96–1.11)	1.02 (0.95–1.11)
Mean tender joint count	0.99 (0.95–1.03)	1.01 (0.96–1.06)	1.03 (0.97–1.09)

Main analysis includes *n* = 139 case-control clusters, sensitivity analysis I *n* = 94 case-control clusters (cases with validated events), sensitivity analysis II *n* = 98 case-control clusters (observation time of at least 12 months)

Matching criteria were sex, age (+/- 5 years), rheumatoid arthritis duration (+/-3 years), date of enrolment (+/- 2 years) and observation time

The multivariable regressions included adjustment for smoking, rheumatoid factor, chronic obstructive pulmonary disease, tuberculosis/chronic viral infections and matching variables

*DAS28 and inflammation marker categories were investigated by categorizing the values for each month according to the categories described in the exposure definition. The number of months with elevated values was then counted and divided by the total observation time in months (first for 12 months and secondly for the entire observation time). This corresponds to the proportion of elevated values

Abbreviations: CI: confidence interval, CRP: C-reactive protein, DAS28: disease activity score based on 28 tender and swollen joint count, ESR: erythrocyte sedimentation rate, OR: Odds ratio

### Sensitivity analyses

Characteristics of cases with a validated ILD diagnosis (sensitivity analysis I) and with an observation time of more than 12 months (sensitivity analysis II) were comparable to the characteristics of all cases. Exceptions were a lower number of obese patients and a lower number of patients treated with csDMARDs in sensitivity analysis I and a higher percentage of patients with rheumatoid nodules in sensitivity analysis II (supplementary Table 6).

Estimates and confidence intervals were comparable to those of the main analysis (Table 3). The models without adjustment for matching variables (supplementary Table 3) were comparable to the models with the respective adjustment (Table 3), but in both sensitivity analyses higher DAS28-ESR was significantly associated with ILD incidence. Excluding patients diagnosed by x-ray (*n* = 11) from sensitivity analysis I resulted in comparable estimates (supplementary Table 7).

## Discussion

This study explored the relationship between the disease activity score DAS28, its individual components, especially the systemic inflammation markers ESR and CRP, and the onset of ILD in individuals with pre-existing RA. Although the composite score DAS28 based on either ESR or CRP did not exhibit a significant relationship with the development of ILD, a notable association was observed with systemic inflammation markers, particularly with persistently elevated levels of ESR and CRP throughout the observation in the RABBIT register. The other components of the DAS28, patient’s global health, tender and swollen joint count, were not associated with a higher odds for ILD.

Interstitial lung diseases are rare, but serious extra-articular manifestations in patients with RA contributing to morbidity and premature mortality. Their incidence is low with reported rates between 0.1% and 7.9% [3, 38–40]. The broad range of reported ILD incidence may be caused by multiple factors, including the type of data source utilized, the nature of the underlying data, associated inclusion criteria of RA patients, the definition of ILD but also the indication for the diagnostic workup (incidental finding vs. ILD screening), and explicit screening methodologies employed for ILD detection. In our cohort, data is collected in daily rheumatology care with no dedicated ILD screening. We observed an ILD incidence of 0.8%. Consistent with other studies [38, 40], the ILD incidence in our cohort remained relatively stable over the investigated time period between 2001 and 2021.

The RABBIT register consists of patients with established RA. The average age in our cohort was around 57 years, three out of four patients were female, the mean duration of RA was nine years and two thirds of patients were RF positive. However, in the subgroup of patients that were diagnosed with ILD during follow-up, we observed a predominance of factors that are associated with a higher ILD risk [3, 14, 15, 41, 42]. Compared to the entire RABBIT cohort, patients with ILD were more often male, had a higher age at enrolment, and a higher frequency of positive RF. In addition, more patients were multimorbid and smokers (current or former) and there were more patients with comorbidities such as obesity, chronic viral infections (e.g., hepatitis C), tuberculosis and chronic obstructive pulmonary disease.

Besides these well described risk factors, several studies have demonstrated associations between RA-ILD and either disease activity or systemic inflammation or both of them. In a meta-analysis, higher levels of ESR and CRP were significantly correlated with RA-ILD [43]. However, the included studies were heterogeneous and mainly rated as having low quality. Most of them investigated inflammation markers at the time of ILD diagnosis, and important information on the timing of data collection and whether ILD was incident or prevalent was lacking. Of note, in a previous analysis of RA patients with prevalent ILD, our group found that a majority of ILD patients (79%) was in moderate to high disease activity measured by DAS28 [44]. And another study showed that the disease activity measured by DAS28-ESR was significantly associated with the (radiological quantitative) severity of ILD [45].

Since higher levels of disease activity or systemic inflammation might have been caused by the ILD itself [46, 47], a more comprehensive and systematic approach is needed to disentangle the relationship between disease activity/ systemic inflammation and the development of ILD. It is essential to make precise assumptions regarding the time of ILD diagnosis and time points of measuring relevant scores and biomarkers. In this analysis, we have therefore decided to look at these parameters from different angles: (I) we have not only investigated the composite score of disease activity DAS28, but also its single components, (II) these factors were investigated as mean continuous values but also in terms of elevated levels, (III) factors were analysed at different time periods prior to ILD and (IV) we adjusted for relevant confounders based on a DAG.

In our study, we found a 3.0- and 3.7-fold increased odds for ILD in patients with persistently elevated systemic inflammation measured by ESR and CRP. This is in line with a very early finding from the Rochester cohort including 582 patients with RA of which 46 patients developed RA-ILD. The analysis revealed an HR of 3.52 (95% CI 1.94–6.38) for patients having at least three times values of ESR ≥ 60 mm/h during their observation time of a mean of 16 years [3]. Differences in ESR levels of RA patients that developed ILD (*n* = 52) compared to those that did not develop ILD (*n* = 1 408) seem to be already obvious in early RA. In the ERAS (Early RA Study) inception cohort from the United Kingdom in which patients with less than two years of RA symptoms and no prior DMARD treatment were included, higher ESR at the baseline visit, but not higher DAS28-ESR was significantly associated with ILD onset [28]. In contrast, an association between time-varying ESR levels > 28 mm/h and incident ILD was not confirmed in a United States (US) study of electronic medical records including 5 817 RA patients without and 205 with ILD [26]. In the same study, elevated CRP levels of > 5 mg/L were significantly associated with a 2.4-fold increase in ILD risk. The US cohort Brigham RA Sequential Study (BRASS) comes to contradictory results with regard to CRP. A nested case-control study within BRASS showed a significant association for high-positive CRP defined as ≥ 10 mg/L with an adjusted OR of 2.61 (95% CI 1.21–5.64) compared to CRP < 3 mg/L [24], but a previous analysis of this cohort neither found an association for high-positive nor for continuous CRP [22]. With regard to DAS28, both BRASS studies revealed a significant 2- to 4-fold higher risk between moderate to high disease activity and ILD onset [22, 24]. The latter study focused on the prediction of ILD by DAS28, and the main results were confirmed by multiple sensitivity analyses.

In our study, other individual components of the DAS28 apart from markers of systemic inflammation were not associated with ILD development. For the swollen joint count, no significant association was revealed by two studies [3, 26]. Furthermore, we are not aware of any study with adjusted data on tender joint count or patient’s global health.

All of the studies that analysed the impact of disease activity or inflammation on ILD onset have their strengths and shortcomings. Comparable to our study, the study by Sparks et al. focused on disease activity as predictor of incident ILD [22]. However, other outcomes such as ESR, joint counts and patient’s global health were not analysed. Others only focused on ESR [3, 28] or on ESR and CRP [26]. Most of the studies described above aimed to investigate general risk factors for ILD in patients with RA, and adjustments of the regression models were based on univariate *p*-values [28] or on established risk factors such as age, sex and smoking [3, 22, 24, 26]. Other important factors were not always included into the models, e.g., autoantibody status only by three studies [22, 24, 26] and comorbidities only by two studies [24, 26]. Since the recording of ACPA-levels in the RABBIT register started in 2007 and consequently does not cover the whole observation time of our study, we did not analyse ACPA-levels.

As secondary outcome we investigated antirheumatic treatment prior to ILD. At the time of ILD diagnosis, ILD cases were more likely to be in the second line of b/tsDMARD treatment compared to matched controls, and furthermore T-cell and B-cell therapy were used more often in ILD cases. Since a channeling bias cannot be ruled out, we have refrained from calculating incidence rates under different therapies. Our finding of a higher use of T-cell and B-cell therapy in RA patients that develop ILD is in line with other studies, e.g., in German claims data, a notable rise in the utilization of T-cell and B-cell therapy, alongside tocilizumab and JAKi use, was observed at the time of ILD diagnosis [38]. In US claims data, incidence rates for ILD were highest in patients that have received B-cell therapy [48, 49]. However, adjusted analysis with adalimumab as comparator group did not reveal a higher ILD risk with B-cell therapy in one of the studies [48].

The strengths of our study are the availability of well-monitored follow-up data of the large RABBIT cohort with a small proportion of missing values [30]. Further, the long observation times of patients enrolled in RABBIT enabled us to select a sufficiently large group of incident RA-ILD cases. A limitation is that in RABBIT AEs are based on physician reports. Even though participating rheumatologists are asked to provide outpatient records or hospital discharge letters for SAEs, specific information was not always available even after request. Since ILD symptoms are rather unspecific, a sensitivity analysis was restricted to ILD events verified by imaging, whereas a differentiation between CT and HRCT on the basis of the provided information was not possible. The results of this sensitivity analysis supported the findings of our main analysis in which all reported events were included. Since x-ray is not sensitive and specific enough for the diagnosis of ILD [8], we performed an additional analysis by excluding those patients from sensitivity analysis I to ensure that our results are not distorted by misdiagnosis due to imprecise imaging. This did not change the results. As RA-ILD often remains asymptomatic for a long time [12], differentiating the cause of inflammation may be difficult. Therefore, we analysed patients with an observation time of more than 12 months (sensitivity analysis II). With this approach, we aimed to reduce the possibility that the inflammation is essentially driven by the pre-existing, subclinical ILD and instead investigated the influence of RA disease activity on the development of ILD. Nevertheless, ILD develops slowly, and without dedicated screening it may not be detected for a long time. In our analysis, we cannot rule out that the ILD developed years before the reporting to RABBIT, possibly even before enrolment into the register. Information about disease activity/inflammation can only be analysed from the time of enrolment, hence we cannot make any assumptions of the time before. In addition, we saw a tendency towards higher DAS28 levels being associated with an increased risk of ILD, but this association was not significant in every model. This could be due to the fact that case-control studies have a reduced power [50], which may limit our results. A further limitation of our study is that the results may not be generalizable to the entire RA population, especially not to patients with early RA. Due to the inclusion criteria of the RABBIT register, the cohort consists of patients with established RA disease. Cases of RA-ILD could only be included in the analysis when reported. Consequently, our results might not be applicable for RA-ILD cases that were not significant enough to be reported as AE.

## Conclusion

Systemic inflammation plays a central role in the development of ILD in patients with RA. We found that persistently elevated ESR and CRP levels were associated with a higher chance of developing ILD. Therefore, rheumatologists should not only monitor composite disease activity scores such as DAS28, but also pay attention to the time-varying levels of ESR and CRP. Tight control of systemic inflammation may thus not only improve the outcome of RA itself, but also help prevent development of RA-ILD. Furthermore, standardised, routine ILD screening is crucial for the early detection of RA-ILD.

## Electronic supplementary material

Below is the link to the electronic supplementary material.

Supplementary Material 1

## Data Availability

The datasets analysed during the current study are not publicly available.

## Abbreviations

ACPA: Anti-citrullinated protein antibodies
AE: Adverse event
BAL: Bronchoalveolar lavage
B-cell: B-cell-targeted therapy
bDMARD: Biologic disease-modifying antirheumatic drug
BRASS: Brigham rheumatoid arthritis Sequential Study
CI: Confidence interval
CRP: C-reactive protein
csDMARD: Conventional synthetic disease-modifying antirheumatic drug
CT: Computed tomography
DAG: Directed acyclic graph
DAS28: Disease activity score based on 28 tender and swollen joint count
DMARD: Disease-modifying antirheumatic drug
e.g.: *exempli gratia*; for example
ERAS: Early rheumatoid arthritis study
ESR: Erythrocyte sedimentation rate
HRCT: High-resolution computed tomography
ICD-10: International classification of diseases, 10th revision
ILD: Interstitial lung disease
JAKi: Janus kinase inhibitors
IL6i: Interleukin-6 inhibitors
MedDRA: Medical dictionary for regulatory activities
mg/l: Milligramm per litre
mm/h: Millimeters per hour
MUC5B: Mucin-5B
MTX: Methotrexate
OR: Odds-ratio
RA: Rheumatoid arthritis
RABBIT: Rheumatoid Arthritis: Observation of Biologic Therapy
RF: Rheumatoid factor
SAE: Serious adverse event
SAS: Statistical analysis system
T-cell: T-cell co-stimulation modulator
TNFi: Tumor necrosis factor inhibitors
tsDMARD: Targeted synthetic disease-modifying antirheumatic drug
US: United States
vs.: versus
