# Supplementary material for: Impact of systemic inflammation and disease activity on the incidence of interstitial lung disease in patients with rheumatoid arthritis – a nested case-control study within the German biologics register RABBIT
Source: Arthritis Res Ther. 2024 Dec 5;26:209. doi: 10.1186/s13075-024-03449-9 (PMC11619653; doi:10.1186/s13075-024-03449-9)
Supplement: Supplementary file 1 — Supplementary Material 1 [file 13075_2024_3449_MOESM1_ESM.pdf]

Impact of systemic inflammation and disease activity on the incidence of interstitial lung disease in patients with rheumatoid arthritis – a nested case-control study within the German biologics register RABBIT

Ronja Ramien, Tatjana Rudi, Rieke Alten, Andreas Krause, Matthias Schneider, Martin Schaefer, Anja Strangfeld, Yvette Meissner

Supplement

Supplementary figure 1: Design of the case-control study.

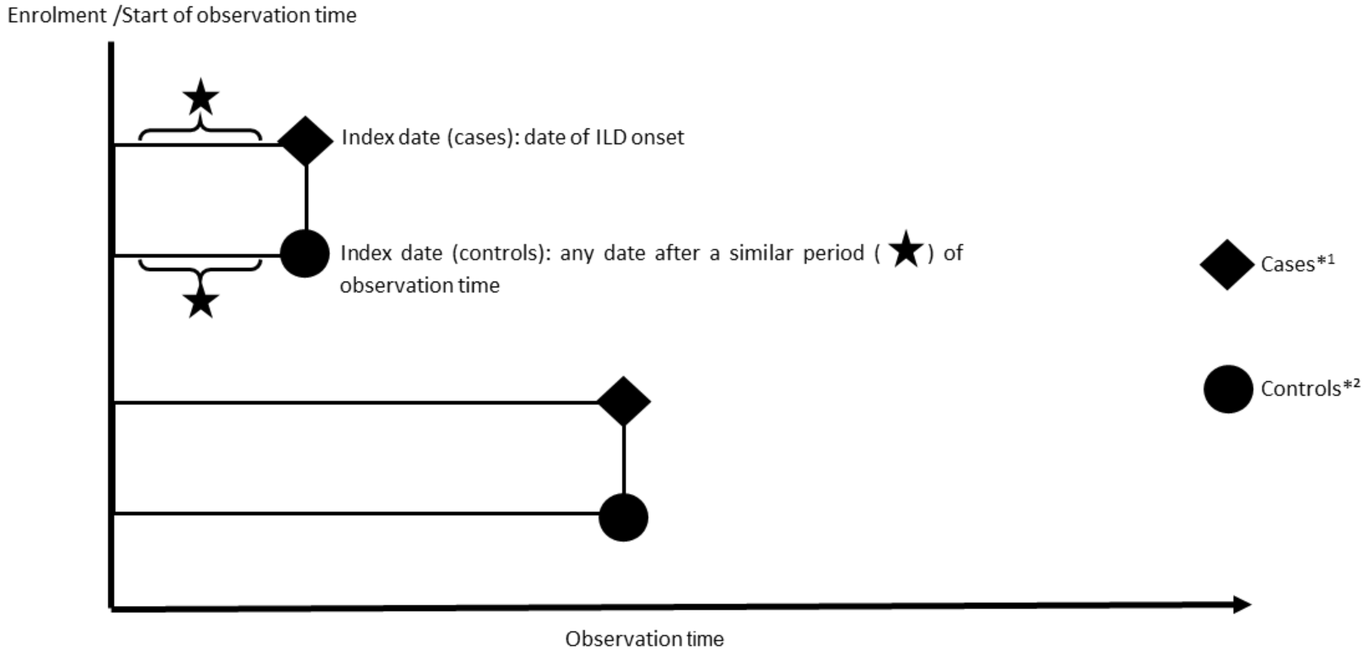

*Cases and controls have a similar date of enrolment (+/- 2 years). The index date of the cases (*1) is equivalent to the date of the diagnosis of the ILD, the index date of the controls (*2) is any date after a similar period of observation time.*

Abbreviations: ILD: interstitial lung disease.

Supplementary figure 2: Directed Acyclic Graph depicting confounder selection.

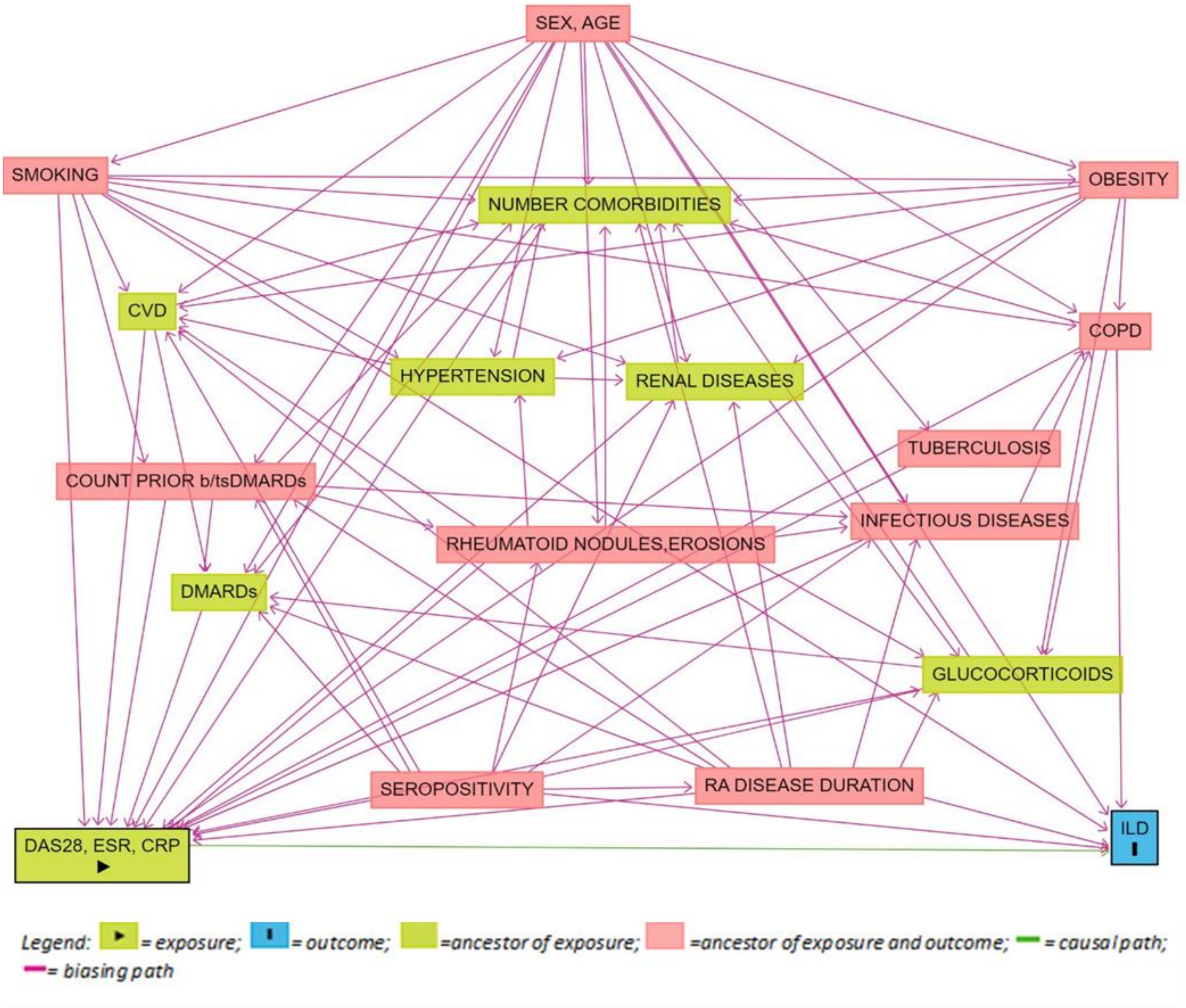

Exposure of interest: DAS28, ESR and CRP.

Confounders for minimal sufficient adjustment set for estimating the total effect of DAS28, ESR, CRP on interstitial lung disease: Age, sex, disease duration of rheumatoid arthritis, smoking (ever/never/missing), seropositivity defined as positive rheumatoid factor, COPD, tuberculosis/chronic viral infections.

Explanations: DAS28, ESR and CRP values were measured from baseline until index date, comorbidities were measured from baseline until index date and all other variables were measured at baseline.

Abbreviations: bDMARD: biologic disease-modifying antirheumatic drug, COPD: chronical obstructive lung disease, CRP: C-reactive protein, CVD: cardiovascular diseases, DAS28: disease activity score based on 28 tender and swollen joint count, DMARDs: disease-modifying antirheumatic drugs, ESR: erythrocyte sedimentation rate, ILD: interstitial lung disease, RA: rheumatoid arthritis, tsDMARD: targeted synthetic disease-modifying antirheumatic drug.

Number of comorbidities comprise: arterial hypertension, coronary heart disease, heart failure, history of cerebrovascular events, hyperlipidemia, diabetes, chronic kidney disease, chronic viral infection, chronic liver disease, bronchial asthma, duodenal/ gastric ulcer, chronic obstructive lung disease, other gastrointestinal diseases, degenerative spine disease, degenerative joint disease, osteoporosis, Sjoegren syndrome, psoriasis, fibromyalgia, psychological disorders, latent tuberculosis, lymphoma/leukaemia, malignant neoplasia.

Supplementary figure 3: Incidence proportion of RA-ILD over time.

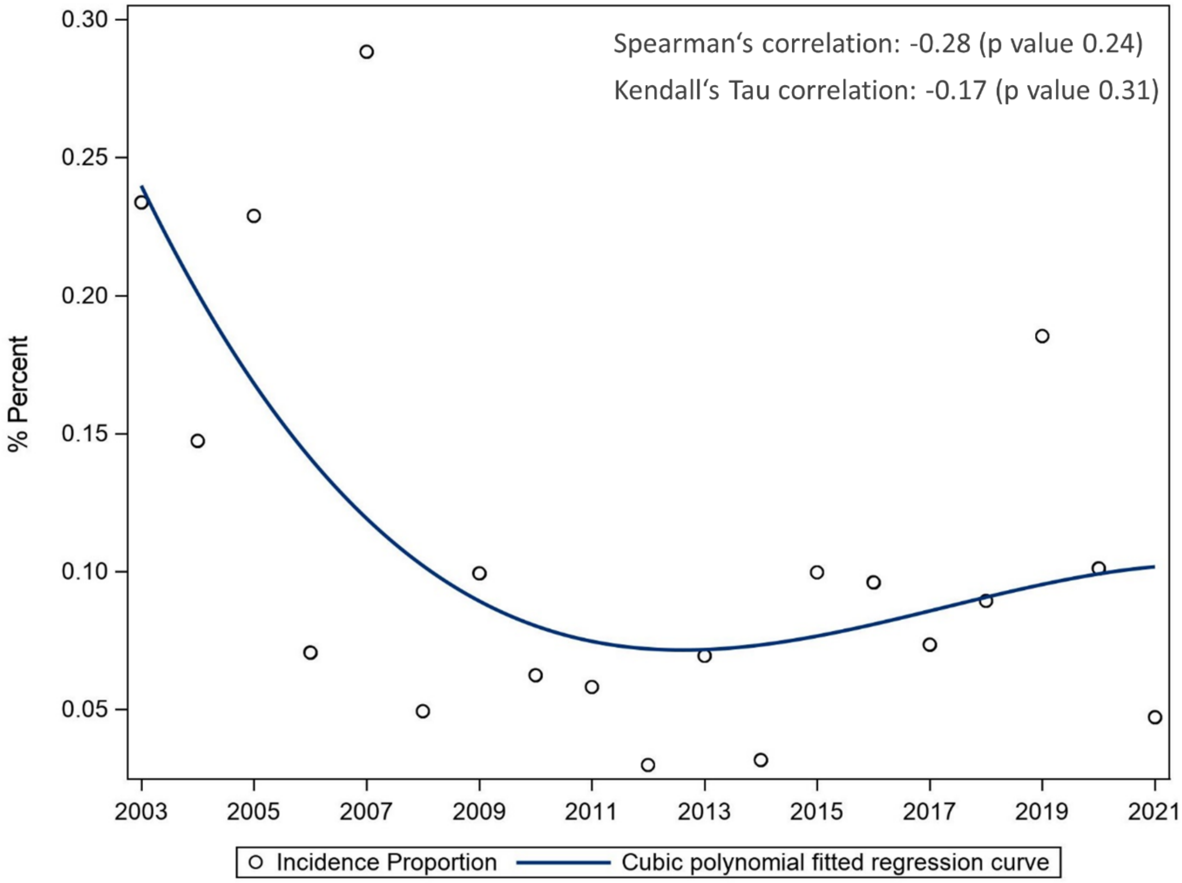

The course of the incidence over time was modelled using cubic polynomial regression.

Supplementary table 1: International Classification of Diseases, 10th Revision (ICD-10) codes for interstitial lung disease and rheumatoid arthritis with interstitial lung disease.

ICD-10 code	Description
**ILD**	
J84.09	Diffuse Alveolar damage (DAD)
J84.1	Pulmonary fibrosis, unspecified
J84.111	Idiopathic interstitial pneumonia (UIP)
J84.112	Idiopathic pulmonary fibrosis (IPF)
J84.113	Idiopathic non-specific interstitial pneumonia (NSIP)
J84.114	Acute interstitial pneumonitis (AIP)
J84.115	Respiratory bronchiolitis interstitial lung disease (RB-ILD)
J84.116	Cryptogenic organizing pneumonia (COP)
J84.117	Desquamative interstitial pneumonia (DIP)
J84.2	Lymphoid interstitial pneumonia (LIP)
J84.8	Other specified interstitial pulmonary disease
J84.9	Interstitial pulmonary disease, unspecified
**RA-ILD**	
J99.0	Respiratory disorders in diseases classified elsewhere
M05.1	Rheumatoid lung disease

Abbreviations: ICD-10: International Classification of Diseases, 10th Revision, ILD: interstitial lung disease, RA-ILD: rheumatoid arthritis with interstitial lung disease.

Supplementary table 2: Results of unadjusted logistic regression analysis for the chance of developing interstitial lung disease.

	Main analysis	Sensitivity analyses	
		I	II
	OR (95% CI)	OR (95% CI)	OR (95% CI)
**Within 12 months prior to index date**			
Mean DAS28-ESR	1.22 (1.04-1.44)	1.26 (1.04-1.54)	1.30 (1.07-1.58)
DAS28-ESR≥3.2 vs. DAS28-ESR<3.2*	1.45 (0.86-2.45)	1.40 (0.75-2.62)	1.69 (0.95-2.99)
Mean DAS28-CRP	1.12 (0.93-1.34)	1.15 (0.93-1.44)	1.23 (0.99-1.53)
DAS28-CRP≥2.9 vs. DAS28-CRP<2.9*	1.04 (0.62-1.74)	1.16 (0.63-2.14)	1.16 (0.66-2.02)
Mean Log ESR	1.77 (1.35-2.31)	1.75 (1.28-2.38)	1.60 (1.18-2.17)
ESR>21 mm/h vs. ESR≤21 mm/h*	2.71 (1.70-4.33)	2.59 (1.49-4.49)	2.32 (1.32-4.08)
Mean Log CRP	1.55 (1.25-1.93)	1.54 (1.20-1.99)	1.57 (1.19-2.06)
CRP≥5 mg/L vs. CRP<5 mg/L*	3.00 (1.82-4.95)	2.76 (1.55-4.92)	2.88 (1.62-5.13)
Mean patient's global health	0.95 (0.86-1.05)	0.97 (0.85-1.09)	0.97 (0.86-1.10)
Mean swollen joint count	1.01 (0.96-1.07)	1.01 (0.94-1.09)	1.02 (0.94-1.10)
Mean tender joint count	1.01 (0.97-1.04)	1.01 (0.97-1.06)	1.04 (1.00-1.09)
**Total observation until the index date**			
Mean DAS28-ESR	1.19 (1.00-1.43)	1.26 (1.02-1.57)	1.27 (1.02-1.58)
DAS28-ESR≥3.2 vs. DAS28-ESR<3.2*	1.32 (0.69-2.54)	1.35 (0.61-3.01)	1.67 (0.79-3.56)
Mean DAS28-CRP	1.07 (0.88-1.30)	1.14 (0.90-1.44)	1.18 (0.93-1.50)
DAS28-CRP≥2.9 vs. DAS28-CRP<2.9*	1.05 (0.56-1.98)	1.15 (0.54-2.46)	1.26 (0.61-2.59)
Mean Log ESR	1.98 (1.46-2.69)	1.93 (1.35-2.76)	1.80 (1.25-2.60)
ESR>21 mm/h vs. ESR≤21 mm/h *	3.25 (1.92-5.52)	2.88 (1.57-5.29)	2.87 (1.46-5.64)
Mean Log CRP	1.64 (1.30-2.07)	1.62 (1.23-2.13)	1.74 (1.27-2.37)
CRP≥5 mg/L vs. CRP<5 mg/L*	3.95 (2.17-7.21)	3.36 (1.68-6.70)	4.31 (2.05-9.07)
Mean patient's global health	0.93 (0.83-1.04)	0.93 (0.81-1.06)	0.95 (0.83-1.09)
Mean swollen joint count	1.02 (0.96-1.08)	1.04 (0.97-1.11)	1.04 (0.96-1.12)
Mean tender joint count	0.99 (0.95-1.03)	1.01 (0.96-1.06)	1.02 (0.97-1.08)

Main analysis included n=139 case-control clusters, sensitivity analysis I n=94 case-control clusters (cases validated events), sensitivity analysis II n=98 case-control clusters (cases with observation time of at least 12 months).

Matching criteria were sex, age (+/- 5 years), RA duration (+/-3 years), date of enrolment (+/- 2 years) and observation time.

**DAS28 and inflammation marker categories were investigated by categorizing the values for each month according to the categories described in the exposure definition. The number of months with elevated values was then counted and divided by the total observation time in months (first for 12 months and secondly for the entire observation time). This corresponds to the proportion of elevated values.*

Abbreviations: CI: confidence interval, CRP: C-reactive protein, DAS28: disease activity score based on 28 tender and swollen joint count, ESR: erythrocyte sedimentation rate, OR: Odds ratio.

Supplementary table 3: Results of adjusted multivariable logistic regression analysis for the chance of developing interstitial lung disease without adjusting for matching variables.

	Main analysis	Sensitivity analyses	
		I	II
	OR (95% CI)	OR (95% CI)	OR (95% CI)
**Within 12 months prior to index date**			
Mean DAS28-ESR	1.21 (1.03-1.43)	1.26 (1.04-1.55)	1.31 (1.08-1.61)
DAS28-ESR≥3.2 vs. DAS28-ESR<3.2*	1.49 (0.87-2.53)	1.51 (0.79-2.86)	1.78 (0.99-3.22)
Mean DAS28-CRP	1.13 (0.94-1.35)	1.18 (0.94-1.48)	1.27 (1.01-1.58)
DAS28-CRP≥2.9 vs. DAS28-CRP<2.9*	1.09 (0.64-1.84)	1.29 (0.68-2.44)	1.25 (0.70-2.23)
Mean Log ESR	1.73 (1.31-2.28)	1.70 (1.23-2.34)	1.58 (1.15-2.17)
ESR>21 mm/h vs. ESR≤21 mm/h*	2.60 (1.61-4.20)	2.47 (1.38-4.41)	2.24 (1.24-4.05)
Mean Log CRP	1.52 (1.22-1.90)	1.48 (1.14-1.93)	1.56 (1.17-2.07)
CRP≥5 mg/L vs. CRP<5 mg/L*	2.87 (1.72-4.78)	2.52 (1.39-4.57)	2.77 (1.52-5.04)
Mean patient's global health	0.93 (0.83-1.04)	0.97 (0.85-1.10)	0.97 (0.85-1.09)
Mean swollen joint count	1.02 (0.97-1.08)	1.02 (0.95-1.09)	1.03 (0.95-1.11)
Mean tender joint count	1.01 (0.97-1.05)	1.02 (0.97-1.07)	1.05 (1.01-1.10)
**Total observation until the index date**			
Mean DAS28-ESR	1.18 (0.98-1.41)	1.27 (1.01-1.57)	1.27 (1.01-1.60)
DAS28-ESR≥3.2 vs. DAS28-ESR<3.2*	1.32 (0.67-2.85)	1.43 (0.63-3.28)	1.74 (0.79-3.83)
Mean DAS28-CRP	1.07 (0.88-1.31)	1.15 (0.90-1.47)	1.20 (0.93-1.54)
DAS28-CRP≥2.9 vs. DAS28-CRP<2.9*	1.10 (0.57-2.12)	1.19 (0.54-2.62)	1.41 (0.69-3.02)
Mean Log ESR	1.91 (1.39-2.62)	1.84 (1.27-2.67)	1.74 (1.18-2.55)
ESR>21 mm/h vs. ESR≤21 mm/h *	3.06 (1.78-5.27)	2.69 (1.42-5.09)	2.64 (1.31-5.34)
Mean Log CRP	1.58 (1.25-2.01)	1.54 (1.16-2.04)	1.68 (1.22-2.33)
CRP≥5 mg/L vs. CRP<5 mg/L*	3.70 (2.00-6.85)	2.97 (1.47-6.01)	3.97 (1.82-8.68)
Mean patient's global health	0.92 (0.82-1.04)	0.93 (0.81-1.08)	0.95 (0.82-1.10)
Mean swollen joint count	1.02 (0.97-1.08)	1.04 (0.97-1.11)	1.04 (0.96-1.12)
Mean tender joint count	0.99 (0.95-1.03)	1.01 (0.96-1.07)	1.03 (0.98-1.09)

Main analysis included n=139 case-control clusters, sensitivity analysis I n=94 case-control clusters (cases validated events), sensitivity analysis II n=98 case-control clusters (cases with observation time of at least 12 months).

Matching criteria were sex, age (+/- 5 years), rheumatoid arthritis duration (+/-3 years), date of enrolment (+/- 2 years) and observation time.

The multivariable regressions included adjustment for smoking, rheumatoid factor, chronic obstructive pulmonary disease, tuberculosis/chronic viral infections.

**DAS28 and inflammation marker categories were investigated by categorizing the values for each month according to the categories described in the exposure definition. The number of months with elevated values was then counted and divided by the total observation time in months (first for 12 months and secondly for the entire observation time). This corresponds to the proportion of elevated values.*

Abbreviations: CI: confidence interval, CRP: C-reactive protein, DAS28: disease activity score based on 28 tender and swollen joint count, ESR: erythrocyte sedimentation rate, OR: Odds ratio.

Supplementary table 4: Missing values at baseline.

	**Cases** **N= 139**	**Controls** **N= 686**	**Remainder of the cohort** **N=17 240**
DAS28-ESR	6 (4.3)	37 (5.4)	1 267 (7.3)
DAS28-CRP	12 (8.6)	41 (6.0)	1 313 (7.6)
ESR	2 (1.4)	25 (3.6)	1 101 (6.4)
CRP	12 (8.6)	40 (5.9)	1 296 (7.5)
Tender joints	0	3 (0.4)	16 (0.1)
Swollen joints	0	2 (0.3)	13 (0.1)
Patient's global health	4 (2.9)	9 (1.3)	160 (0.9)
Rheumatoid factor	1 (0.7)	5 (0.7)	296 (1.7)
Smoking	6 (4.3)	28 (4.1)	1 222 (7.1)

Abbreviations: CRP: C-reactive protein, DAS28: disease activity score based on 28 tender and swollen joint count, ESR: erythrocyte sedimentation rate.

Supplementary table 5: Patients characteristics of not fully matched cases.

	**Not fully matched cases**<br>**N=5**
Age (years), mean (SD)	74.2 (9.7)
Sex, female	5
RA duration (years), mean (SD)	26 (11.1)
Observation time (months), mean (SD)	72 (32.8)
DAS28-ESR	4.0 (1.4)
DAS28-CRP	3.2 (1.1)
ESR (mm/hour), mean (SD)	22.8 (11.1)
CRP (mg/L), mean (SD)	15.8 (16.5)
Tender joints, mean (SD)	5.4 (6.7)
Swollen joints, mean (SD)	2.0 (2.5)
Patient's global health, mean (SD)	3.6 (2.3)
Rheumatoid factor positive	4 (80.0)
Smoking, ever	1 (20.0)
Count of comorbidities*, mean (SD)	5.2 (3.1)
Chronic obstructive pulmonary disease	0
Chronical viral infection*[1]	0
Latent tuberculosis	0

Values are numbers of patients (%) unless otherwise specified.

**Comorbidities comprise arterial hypertension, coronary heart disease, heart failure, cerebral ischemia, hyperlipidemia, diabetes, chronic kidney disease, chronic viral infection, chronic liver disease, bronchial asthma, duodenal/ gastric ulcer, chronic obstructive lung disease, other gastrointestinal diseases, degenerative spine disease, degenerative joint disease, osteoporosis, Sjoegren syndrome, psoriasis, fibromyalgia, psychological disorders, latent tuberculosis, lymphoma/leukaemia, malignant neoplasia.*

*[1]*Chronic viral infection comprise hepatitis B, hepatitis C, human immunodeficiency virus.*

Abbreviations: CRP: C-reactive protein, DAS28: disease activity score based on 28 tender and swollen joint count, ESR: erythrocyte sedimentation rate, SD: standard deviation.

Supplementary table 6: Enrolment characteristics of cases (patients who developed an interstitial lung disease during follow up), their matched controls of sensitivity analysis I+II.

	**Sensitivity analysis I**		**Sensitivity analysis II**	
	**Cases N=94**	**Controls N=467**	**Cases N=98**	**Controls N=481**
**Matching criteria**				
Sex, female	63 (67.0)	312 (66.8)	66 (67.3)	321 (66.7)
Age (years), mean (SD)	62.8 (10.5)	62.0 (10.2)	61.7 (9.7)	60.7 (9.4)
RA duration (years), mean (SD)	9.9 (9.6)	9.8 (9.3)	10.4 (9.6)	10.0 (9.1)
Year of enrolment 2001 - 2005	17 (18.1)	76 (16.3)	30 (30.6)	145 (30.1)
Year of enrolment 2006 - 2010	26 (27.7)	131 (28.1)	29 (29.6)	145 (30.1)
Year of enrolment 2011 - 2015	30 (31.9)	156 (33.4)	30 (30.6)	145 (30.1)
Year of enrolment 2016 - 2021	21 (22.3)	104 (22.2)	9 (9.2)	46 (9.6)
Observation time (months), mean (SD)	39.4 (35.3)	39.2 (35.1)	51.6 (30.3)	51.1 (30.0)
**Unmatched criteria**				
Age at RA onset (years), mean (SD)	52.9 (13.4)	52.2 (13.2)	51.3 (13.0)	50.6 (12.7)
DAS28-ESR, mean (SD)	5.2 (1.4)	5.0 (1.3)	5.3 (1.5)	5.1 (1.3)
DAS28-CRP, mean (SD)	4.0 (1.1)	3.8 (1.1)	4.1 (1.1)	3.9 (1.1)
ESR (mm/hour), mean (SD)	34.5 (22.1)	29.2 (22.9)	33.6 (23.1)	29.5 (21.6)
CRP (mg/L), mean (SD)	18.8 (22.1)	16.4 (22.5)	19.7 (26.3)	17.2 (22.3)
Tender joints, mean (SD)	9.1 (7.2)	8.3 (7.0)	9.8 (7.1)	8.5 (6.9)
Swollen joints, mean (SD)	6.8 (5.6)	6.0 (5.1)	7.3 (5.9)	6.6 (5.3)
Patient’s global health, mean (SD)	5.7 (2.2)	5.9 (2.0)	5.9 (2.2)	5.8 (2.0)
% of full physical function (FFbH), mean (SD)	61.9 (25.2)	62.6 (23.0)	60.6 (26.6)	64.3 (22.8)
Rheumatoid factor positive	77 (81.9)	328 (70.2)	81 (82.7)	352 (73.2)
Rheumatic nodules	26 (27.7)	65 (13.9)	34 (34.7)	70 (14.6)
Erosions of joints	32 (34.0)	195 (41.8)	33 (33.7)	175 (36.4)
Smoking, ever	57 (60.6)	239 (51.2)	62 (63.3)	233 (48.4)
Smoking, never	36 (38.3)	217 (46.4)	33 (33.7)	236 (49.1)
Count of comorbidities*, mean (SD)	3.6 (3.0)	2.6 (2.5)	3.3 (2.8)	2.3 (2.2)
Chronical viral infection[#]	2 (2.1)	2 (0.4)	2 (2.0)	1 (0.2)
Chronic obstructive pulmonary disease	10 (10.6)	31 (6.6)	7 (7.1)	26 (5.4)
Coronary heart disease	13 (13.8)	32 (6.9)	14 (14.3)	33 (6.9)
Chronic kidney disease	10 (10.6)	27 (5.8)	7 (7.1)	21 (4.4)
Arterial hypertension	56 (59.6)	223 (47.8)	63 (64.3)	218 (45.3)
Osteoporosis	29 (30.9)	103 (22.1)	27 (27.6)	95 (19.5)
Latent tuberculosis	6 (6.4)	10 (2.1)	5 (5.1)	8 (1.7)

BMI ≥30 kg/m^2	22 (23.4)	118 (25.3)	29 (29.6)	114 (23.7)
Enrolment therapy: csDMARD	23 (24.5)	151 (32.3)	32 (32.7)	174 (36.2)
Enrolment therapy: TNFi	44 (46.8)	213 (45.6)	44 (44.9)	235 (48.9)
Enrolment therapy: T-cell	7 (7.4)	16 (3.4)	4 (4.1)	11 (2.3)
Enrolment therapy: B-cell	12 (12.8)	27 (5.8)	10 (10.2)	26 (5.4)
Enrolment therapy: IL6i	6 (6.4)	47 (10.1)	6 (6.1)	31 (6.4)
Enrolment therapy: JAKi	2 (2.1)	13 (2.8)	2 (2.0)	4 (0.8)
Count of prior b/tsDMARD, mean (SD)	0.5 (0.9)	0.4 (0.9)	0.4 (0.9)	0.3 (0.7)
Glucocorticoids, < 5 mg/day	40 (42.6)	208 (44.5)	42 (42.9)	198 (41.2)
Glucocorticoids, 5-10 mg/day	40 (42.6)	188 (40.3)	40 (40.8)	199 (41.4)
Glucocorticoids, > 10 mg/day	13 (13.8)	69 (14.8)	16 (16.3)	84 (17.5)

Values are numbers of patients (%) unless otherwise specified.

Main analysis included n=139 case-control clusters, sensitivity analysis I n=94 case-control clusters (cases validated events), sensitivity analysis II n=98 case-control clusters (cases with observation time of at least 12 months).

Abbreviations: B-cell: B-cell-targeted therapy, bDMARD: biologic disease-modifying antirheumatic drug, BMI: body mass index, CRP: C-reactive protein, csDMARD: conventional synthetic disease-modifying antirheumatic drug, DAS28: disease activity score based on 28 tender and swollen joint count, ESR: erythrocyte sedimentation rate, FFbH: Funktionsfragebogen Hannover, IL-6i: Interleukin-6 inhibitors, JAKi: Janus kinase inhibitors, RA: rheumatoid arthritis, SD: standard deviation. T-cell: T-cell co-stimulation modulator, TNFi: tumour necrosis factor inhibitors, tsDMARD: targeted synthetic disease-modifying antirheumatic drug.

**Comorbidities: arterial hypertension, coronary heart disease, heart failure, cerebral ischemia, hyperlipoproteinemia, diabetes, chronic kidney disease, chronic viral infection (*1hepatitis B, hepatitis C, human immunodeficiency virus), chronic liver disease, asthma bronchiale, ulcus duodeni/ventriculi, chronic obstructive lung disease, other gastrointestinal diseases, degenerative spine disease, degenerative joint disease, osteoporosis, Sjoegren syndrome, Psoriasis, fibromyalgia, psychological disorders, latente tuberculosis, lymphoma/leukemia, malignant neoplasia*

Supplementary table 7: Results of adjusted multivariable logistic regression analysis for the chance of developing interstitial lung disease (sensitivity analyses I without cases diagnosed by x-ray).

	sensitivity analyses I **without cases diagnosed by x-ray** **(N=83)**
	OR (95% CI)
**Within 12 months prior to index date**	
Mean DAS28-ESR	1.27 (1.02-1.59)
DAS28-ESR≥3.2 vs. DAS28-ESR<3.2*	1.46 (0.75-2.86)
Mean DAS28-CRP	1.17 (0.91-1.51)
DAS28-CRP≥2.9 vs. DAS28-CRP<2.9*	1.20 (0.60-2.39)
Mean Log ESR	1.72 (1.21-2.45)
ESR>21 mm/h vs. ESR≤21 mm/h*	2.54 (1.36-4.73)
Mean Log CRP	1.45 (1.10-1.91)
CRP≥5 mg/L vs. CRP<5 mg/L*	2.66 (1.40-5.05)
Mean patient's global health	0.95 (0.83-1.09)
Mean swollen joint count	1.04 (0.96-1.12)
Mean tender joint count	1.02 (0.97-1.08)
**Total observation until the index date**	
Mean DAS-ESR	1.24 (0.96-1.58)
DAS28-ESR≥3.2 vs. DAS28-ESR<3.2*	1.27 (0.52-3.09)
Mean DAS28-CRP	1.14 (0.86-1.51)
DAS28-CRP≥2.9 vs. DAS28-CRP<2.9*	1.16 (0.48-2.82)
Mean Log ESR	1.73 (1.15-2.59)
ESR>21 mm/h vs. ESR≤21 mm/h *	2.45 (1.22-4.90)
Mean Log CRP	1.49 (1.10-2.03)
CRP≥5 mg/L vs. CRP<5 mg/L*	2.95 (1.35-6.41)
Mean patient's global health	0.89 (0.77-1.04)
Mean swollen joint count	1.05 (0.97-1.14)
Mean tender joint count	1.02 (0.96-1.08)

Main analysis includes n=139 case-control clusters, sensitivity analysis I n=94 case-control clusters (cases with validated events), sensitivity analysis Ia n=83 case-control clusters (cases of sensitivity analysis I without x-ray).

Matching criteria were sex, age (+/- 5 years), rheumatoid arthritis duration (+/-3 years), date of enrolment (+/- 2 years) and observation time.

The multivariable regressions included adjustment for smoking, rheumatoid factor, chronic obstructive pulmonary disease, tuberculosis/chronic viral infections and matching variables.

**DAS28 and inflammation marker categories were investigated by categorizing the values for each month according to the categories described in the exposure definition. The number of months with elevated values was then counted and divided by the total observation time in months (first for 12 months and secondly for the entire observation time). This corresponds to the proportion of elevated values.*

Abbreviations: CI: confidence interval, CRP: C-reactive protein, DAS28: disease activity score based on 28 tender and swollen joint count, ESR: erythrocyte sedimentation rate, OR: Odds ratio.
